# Biliary Anomalies in VACTERL Syndrome: A Case Report

**DOI:** 10.7759/cureus.66700

**Published:** 2024-08-12

**Authors:** Fatema Mohamed, Umesh Basavaraju

**Affiliations:** 1 Gastroenterology and Hepatology, Aberdeen Royal Infirmary Hospital, Aberdeen, GBR

**Keywords:** biliary tree anomalies, hepatobiliary, biliary, vacterl, vacterl syndrome

## Abstract

The VACTERL/VATER association is a rare congenital disorder characterized by the presence of at least three of its main components: vertebral defects, anal atresia, cardiovascular anomalies, tracheoesophageal fistulas, esophageal atresia, renal anomalies, and limb defects.

The exact cause of the VACTERL association is not fully understood. Most cases occur randomly. However, some research suggests that genetics and environmental factors may play a role.

In addition to the core components of the VACTERL association, affected individuals may also have anomalies beyond the typical features. Biliary anomalies, although not classically included in the definition, have been documented in individuals with VACTERL syndrome, adding more complexity to this condition. Patients with biliary anomalies may present with jaundice, abdominal pain, or poor growth. The presence of biliary anomalies in individuals with VACTERL syndrome can have an impact on their care and outcomes. Detecting and treating these anomalies usually involves a multidisciplinary team. Timely identification and proper management of these bile-related issues are essential to prevent complications such as cholangitis.

## Introduction

VACTERL/VATER association is a rare congenital disorder that is typically defined by the presence of at least three of the following congenital malformations: vertebral defects, anal atresia, cardiac defects, tracheoesophageal fistulas, renal anomalies, and limb abnormalities [1]. Patients may have other congenital anomalies in addition to these clinical features. The VATER association was first named in the early 1970s [1].

Although the exact cause of this illness is unknown, it is believed to be due to a combination of genetic and environmental factors [1], and in at least a subset of patients, there is evidence for familial clustering suggestive of inherited factors [1]. The incidence of biliary anomalies in VACTERL syndrome is not well established; however, recent papers suggest that biliary anomalies can coexist with VACTERL syndrome, adding more complexity to the clinical picture.

VACTERL incidence is estimated at approximately one in 10,000 to 40,000 live-born infants [1,2]. While some of the VACTERL malformations appear early in the embryological period (23-30 days post conception), others occur later [1,2].

The association of biliary anomalies and VACTERL syndrome is a rare coincidence. Here, we report a case of VACTERL syndrome with recurrent abdominal pain due to associated biliary anomalies.

## Case presentation

A 39-year-old lady was referred to the gastroenterology clinic in 2019 with episodic abdominal pain, vomiting, and cholestatic liver enzymes. She denied any dysphagia, indigestion, or weight loss. She had been diagnosed with a partial VACTERL malformation with a previous colostomy for a congenital cloacal anomaly, which was reversed later. She is a chronic smoker, and she drinks alcohol very rarely.

In 2016, she was complaining of altered bowel habits with alternating constipation and diarrhea. Celiac serology and fecal calprotectin were negative, and she was diagnosed with irritable bowel syndrome.

Since 2013, she has had a mildly raised gamma-glutamyl transferase (GGT), with a range of 100 to 200 units/L. The chronic liver disease screen was checked, and it was negative. An abdominal ultrasound scan in 2015 showed a normal liver and biliary system with no evidence of gallstones, although the portal vein appeared slightly distended.

In 2019, her liver enzymes started to worsen, and her alkaline phosphatase (ALP) began to rise. Her examination revealed bilateral upper limb deformities that are part of her VACTERL syndrome. She also had scarring on her abdomen from a previous surgery in her childhood. Her abdomen was soft and non-tender, with no masses. She had no stigmata of chronic liver disease. Abdominal ultrasound was done, and it showed mild intrahepatic duct dilatation while the common bile duct (CBD) appeared normal in caliber. At the bifurcation of the hepatic duct, there was a prominence, but no cause was identified. The gallbladder was thin and calculi-free.

**Figure 1:**
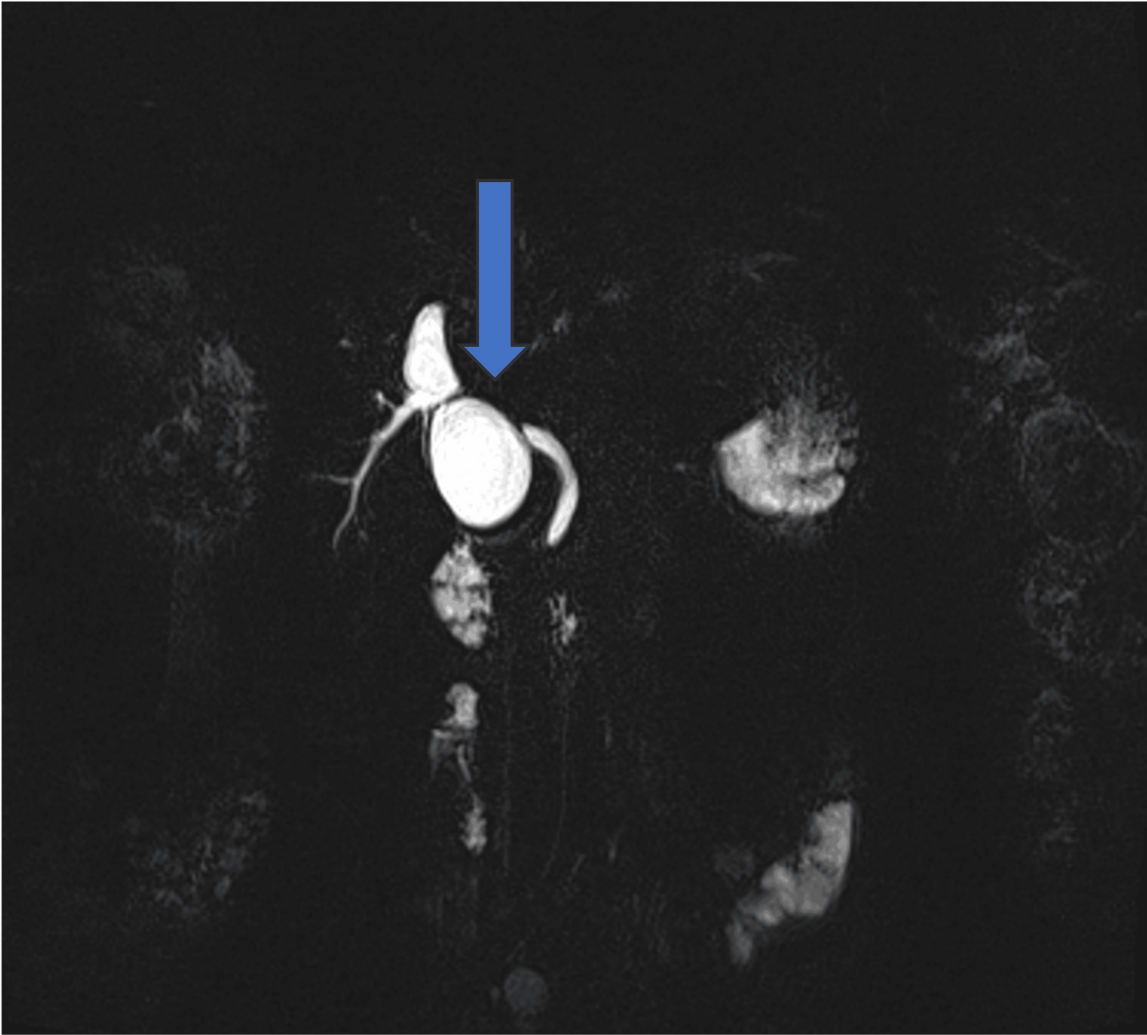
Magnetic resonance cholangiopancreatography (MRCP) image A short stricture in the common hepatic duct and the neck of the gallbladder was in contact with that area.

Magnetic resonance cholangiopancreatography (MRCP) was done on 03/07/2020, which showed intrahepatic biliary dilatation in both lobes of the liver. It appeared to be caused by a short stricture in the common hepatic duct (CHD). The neck of the gallbladder was in contact with that area. The CBD was normal in caliber, measuring around 5 mm. The liver appeared to be normal (Figure 1).

The case was discussed within the hepatobiliary multidisciplinary team (MDT), and the plan was to consider the IgG4 level and endoscopic retrograde cholangiopancreatography (ERCP) with a spyglass for further assessment of the stricture. ERCP was delayed due to COVID-19 and the restriction of the service during the pandemic. Her liver enzymes improved significantly during that time, and there were no signs of jaundice.

Another MRCP was requested for further assessment of her biliary system before deciding about the need for the ERCP. MRCP was done on 27/6/2023, and it showed no significant change in the biliary tree appearances with persisting presumed common hepatic duct stricture.

**Figure 2:**
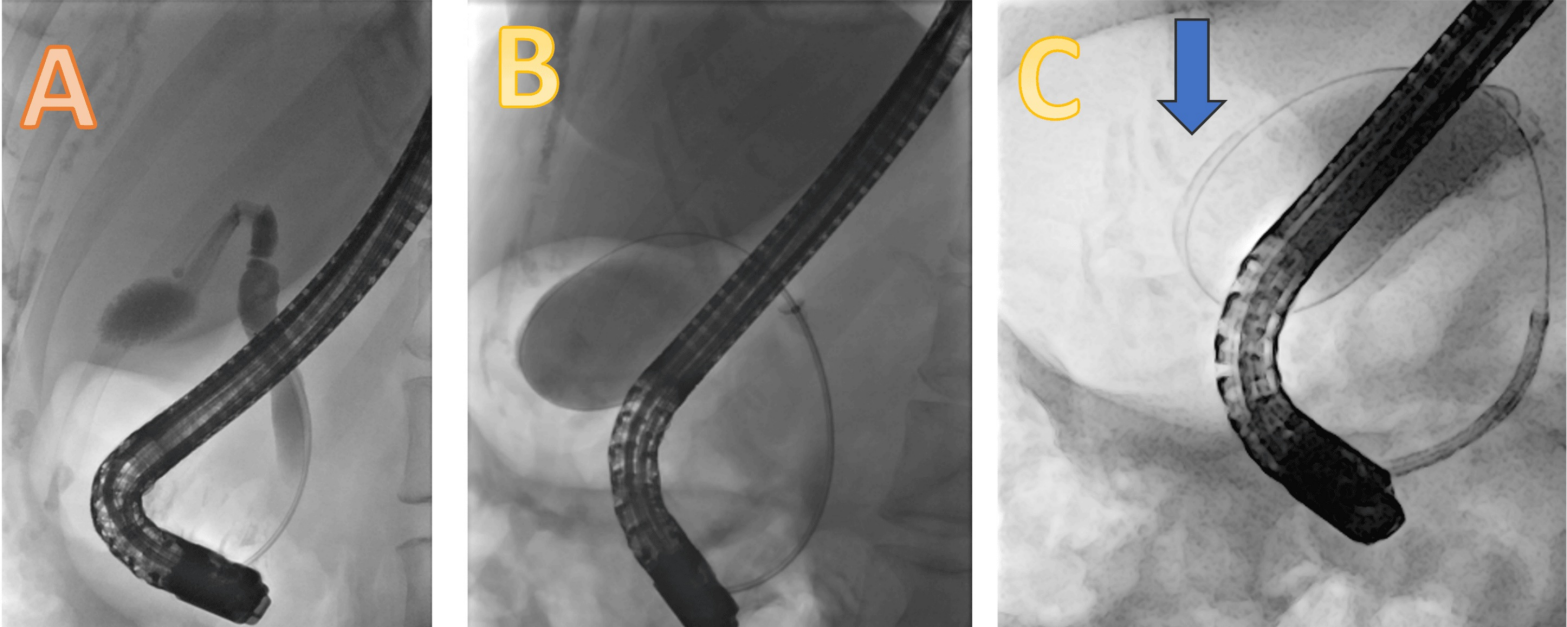
Endoscopic retrograde cholangiopancreatography images A: demonstrate normal lower CBD with no visualization of the CHD or IHD; B: demonstrate the wire going into the gallbladder; C: the blue arrow demonstrate the wire coiling inside the gallbladder CBD: common bile duct; CHD: common hepatic duct; IHD: intrahepatic duct

**Figure 3:**
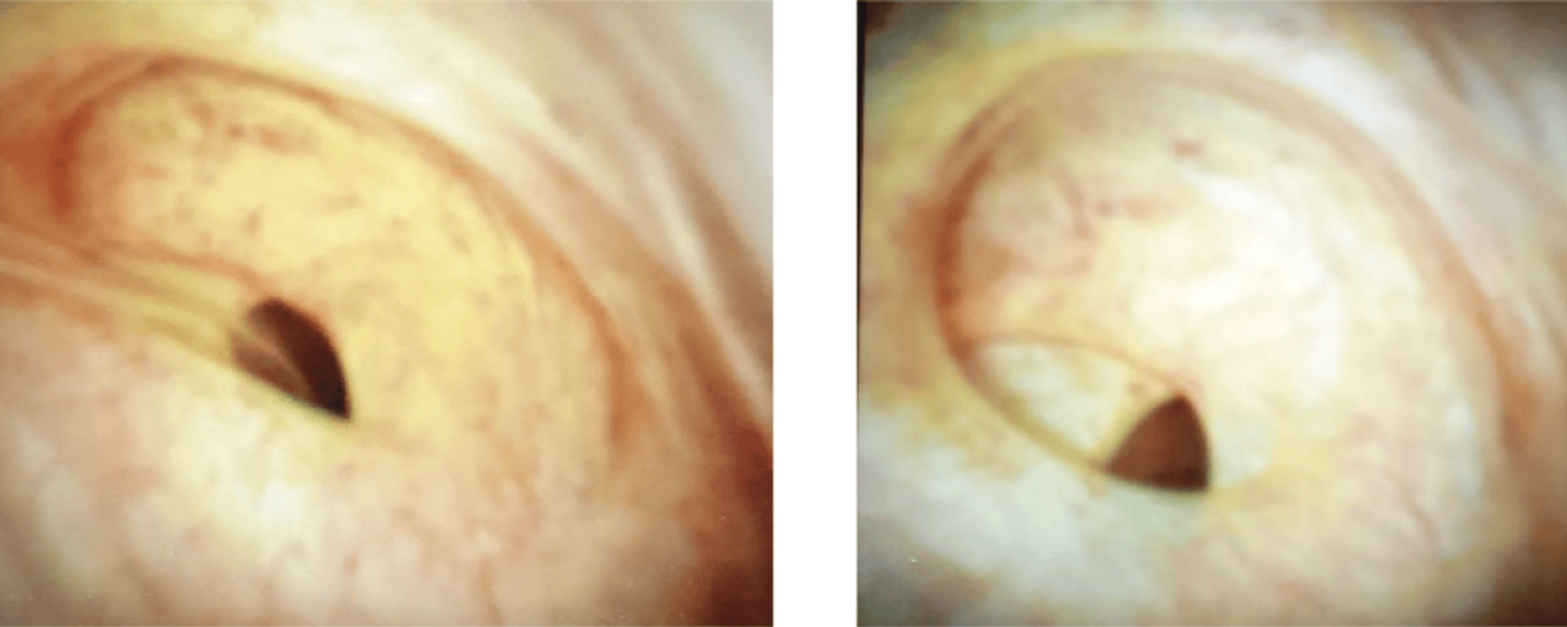
Cholangioscopy images An anomalous anatomy with the bile duct directly leading into the cystic duct.

**Figure 4:**
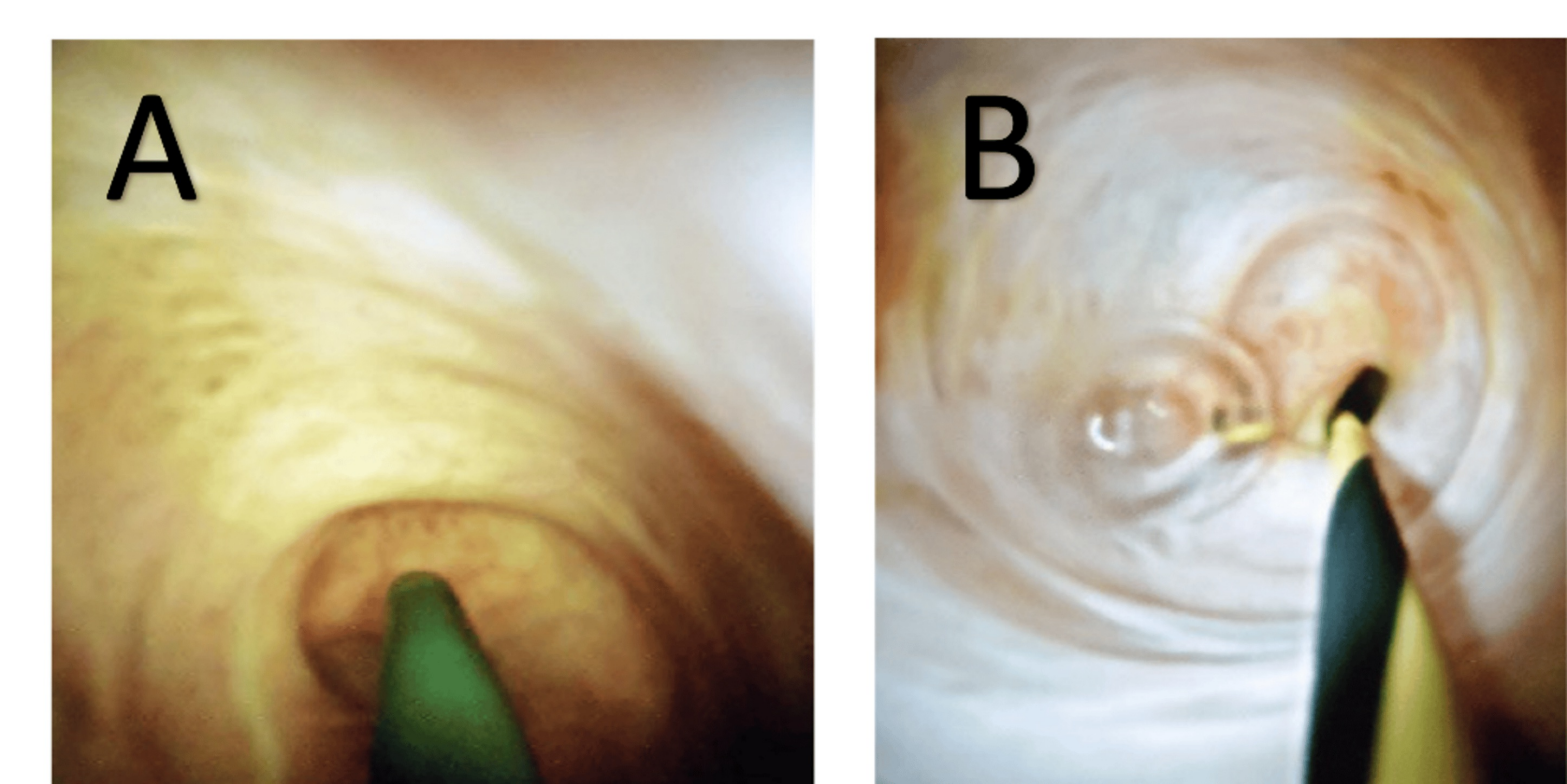
Cholangioscopy images A: attempt to insert the wire into the intrahepatic bile ducts; B: the catheter couldn’t be inserted beyond that area despite multiple attempts using the wire under direct visualization

She underwent an ERCP in August 2023, which showed a normal lower CBD with no visualization of the CHD or intrahepatic duct (IHD). The wire kept going into the gallbladder (Figure 2). Cholangioscopy was done, which showed that there is an anomalous anatomy with the bile duct directly leading into the cystic duct (Figure 3). The cholangioscope catheter couldn’t be inserted beyond that area despite multiple attempts and using the wire under direct visualization (Figure 4).

The case was discussed in the hepatobiliary MDT, and a hepatobiliary iminodiacetic acid (HIDA) scan was requested for further evaluation of the biliary tree before deciding about the need for any surgical intervention.

The patient declined to undergo further diagnostic testing. She expressed a preference to closely monitor her symptoms while her condition remains stable. However, if she experiences a worsening of symptoms, such as the development of jaundice or cholangitis, we will promptly refer her to surgical specialists for comprehensive evaluation and management.

## Discussion

VACTERL/VATER association is defined by the presence of at least three of the following congenital malformations: vertebral defects, anal atresia, cardiac defects, tracheoesophageal fistulas, renal anomalies, and limb abnormalities [1,2]. The incidence is estimated at approximately one in 10,000 to one in 40,000 live births [1,2]. The diagnosis is clinical, requiring the exclusion of similar conditions such as Baller-Gerold syndrome, CHARGE syndrome, and others [1]. The etiology remains largely unknown due to clinical and causal heterogeneity and the sporadic nature of the disorder, but new genetic research methods hold promise for a better understanding [1]. The vast majority of VACTERL cases are sporadic; however, single or multiple malformations associated with VACTERL are observed in first-degree relatives, suggesting an inherited component in a subset of patients [3]. Although the exact cause of the VACTERL association is still unknown, it is believed to be a combination of environmental and genetic factors [1]. Researchers have linked specific gene mutations in the FGF8 gene to this disorder [4]. Additionally, they identified invasive assisted reproductive techniques, primiparity, pregestational overweight and obesity, a lack of folic acid supplement use, and smoking as maternal risk factors involved in the etiology of VACTERL syndrome [5].

Antenatal diagnosis is challenging, and management typically involves surgical correction of specific anomalies in the immediate postnatal period, followed by long-term medical care. The prognosis can be positive if optimal surgical correction is achievable, and patients generally do not have neurocognitive impairment [1].

While biliary abnormalities are not officially considered part of the VACTERL syndrome diagnosis criteria, some studies have shown that they can coexist with this syndrome [2]. In a published case report, a one-day-old female newborn with VACTERL syndrome presents with a cardiac aortopulmonary window (APW) combined with a choledochal cyst [6]. Another case report by Lugo-Vicente in 2009 described a case of double cystic duct in a child with VACTERL syndrome [7].

In a report written by Yoon Y et al. in 2008, a 12-year-old girl with VACTERL syndrome was complaining of abdominal pain. Her investigations showed an abnormal intrahepatic bile duct confluence, which was present as three bile ducts draining directly into the neck of the gall bladder. She was treated with cholecystectomy and choledochojejunostomy [2].

In our case, anomalies in the biliary tree resulted in difficulties in bile drainage, leading to abdominal pain and cholangitis. While the initial diagnosis of biliary anomalies typically involves non-invasive imaging modalities like ultrasound and MRCP, these methods may not always help in providing the diagnosis. There are several advantages to using cholangioscopy for detecting and managing biliary anomalies. It enables direct visualization of the biliary tree, providing detailed images that can aid in identifying and characterizing anomalies that may not be apparent on other imaging modalities. Our case shows that cholangioscopy can be a valuable tool in assessing the biliary anomalies in VACTERL syndrome. Further research and clinical experience will help define its role more clearly in this context.

## Conclusions

In conclusion, the incidence of biliary anomalies in VACTERL syndrome remains not well established, but their presence can significantly complicate the clinical picture. Healthcare providers need to be aware of anomalies in VACTERL association cases, especially when patients experience abdominal pain, elevated liver enzymes, or recurrent cholangitis. Taking an approach that considers the possibility of biliary problems can lead to better outcomes and quality of life for those with VACTERL associations. Our case report highlights the critical role of cholangioscopy in diagnosing these anomalies, offering direct visualization of the biliary tree and revealing configurations not detectable through conventional imaging. This advanced endoscopic technique helps guide precise diagnosis and management. A multidisciplinary approach involving surgeons, gastroenterologists, hepatologists, and interventional endoscopists is essential for optimal care in these complex cases. More research and long-term studies are needed to enhance our understanding of the underlying mechanisms and improve outcomes.
